# Intranasal Delivery of Recombinant AAV Containing BDNF Fused with HA2TAT: a Potential Promising Therapy Strategy for Major Depressive Disorder

**DOI:** 10.1038/srep22404

**Published:** 2016-03-03

**Authors:** Xian-cang Ma, Peng Liu, Xiao-ling Zhang, Wen-hui Jiang, Min Jia, Cai-xia Wang, Ying-ying Dong, Yong-hui Dang, Cheng-ge Gao

**Affiliations:** 1 Department of Psychiatry, First Affiliated Hospital of Xi’an Jiaotong University Health Science Center, Xi’an, China; 2 College of Medicine & Forensics, Xi’an Jiaotong University Health Science Center, Xi’an, China; 3 Department of CT/MRI, Shaanxi Provincial People’s Hospital, Xi’an, China; 4 Xi’an Ankang Hospital, Xi’an, China; 5 Key Laboratory of the Health Ministry for Forensic Medicine, Xi’an Jiaotong University Health Science Center, Xi’an, China; 6 Key Laboratory of Environment and Genes Related to Diseases of the Education Ministry, Xi’an Jiaotong University Health Science Center, Xi’an, China

## Abstract

Depression is a disturbing psychiatric disease with unsatisfied therapy. Not all patients are sensitive to anti-depressants currently in use, side-effects are unavoidable during therapy, and the cases with effectiveness are always accompanied with delayed onset of clinical efficacy. Delivering brain-derived neurotrophic factor (BDNF) to brain seems to be a promising therapy. However, a better approach to delivery is still rudimentary. The purpose of our present work is to look for a rapid-onset and long-lasting therapeutic strategy for major depressive disorder (MDD) by effectively delivering BDNF to brain. BDNF, fused with cell-penetrating peptides (TAT and HA2), was packaged in adenovirus associated virus (AAV) to construct the BDNF-HA2TAT/AAV for intranasally delivering BDNF to central nervous system (CNS) via nose-brain pathway. Intranasal administration of BDNF-HA2TAT/AAV to normal mice displayed anti-depression effect in forced swimming test when the delivery lasted relatively longer. The AAV applied to mice subjected to chronic mild stress (CMS) through intranasal administration for 10 days also alleviated depression-like behaviors. Western-blotting analysis revealed that BDNF-HA2TAT/AAV nasal administration enhanced hippocampal BDNF content. These results indicate intranasal administration of constructed BDNF-HA2TAT/AAV exerts anti-depression effect in CMS mice by increasing hippocampal BDNF, suggesting that this strategy holds a promising therapeutic potential for MDD.

Major depressive disorder (MDD) is affecting approximately 10% of the world population with lifetime prevalence up to 17%^1^. The neuropsychiatric symptoms of depression include depressed mood, markedly diminished interest or pleasure, insomnia or hypersomnia, feelings of worthlessness or excessive or inappropriate guilt and recurrent thoughts of death which bring a lot of troubles to the victims^2^,^3^, and the prevalence of depression in women is two times higher than that in men^4^. Despite its considerable impact, the understanding of depression is rudimentary^5^. Correspondingly, the clinical therapy is less satisfactory than expected. Not all patients are sensitive to the anti-depressants currently in use, side-effects are unavoidable during therapy, and the effective cases always are accompanied with delayed onset of clinical efficacy^6^.

The brain-derived neurotrophic factor (BDNF) is a member of the neurotrophins family (NTs) which is essential for the development of the CNS and for neuronal plasticity^7^. Throughout development, BDNF serves as a signal for proper axonal growth. BDNF also has vital functions in the proper development and survival of dopaminergic, GABAergic, cholinergic, and serotonergic neurons^8^. BDNF depletion has been demonstrated to be responsible for some psychiatric disorders, especially depression^9^. The hippocampal BDNF level in patients was found to be decreased in postmortem study. The BDNF expression could be enhanced after anti-depressant treatment^10^. Besides, implanting sustained-release polymers (alginate microspheres) containing BDNF into dorsal hippocampus of rats could obtain antidepressant-like behavioral effects^11^. Therefore, delivering BDNF to the brain, especially the hippocampus, seems to be a promising therapy to MDD.

Diverse approaches have been employed for BDNF delivery into brain, but the outcomes are less satisfactory than expected. Peripheral delivery may face the obstruction of blood brain barrier (BBB). Some other more direct means have been attempted, including microinjection^12^, intracerebroventricular injection^13^ and use of minipumps^14^. However, these means of delivery may cause tissue damage, and the bioavailability and efficiency would be more or less affected by blood cerebrospinal fluid (BCSF)^15^. Anyway, these methods may be unavailable for human clinical use due to their invasive nature.

In recent years, nose-brain pathway is emerging as an alternative for delivering drugs to brain^16^,^17^,^18^. The adenovirus associated virus (AAV), a single-stranded DNA virus with an icosahedral capsid, is a promising vector for gene transferring *in vivo*^19^. The transactivator of transcription (TAT) protein from human immunodeficiency virus (HIV)-1, an 11-amino acid peptide, is an excellent candidate for protein transduction which could fuse with other proteins to improve their penetration^20^,^21^. The HA2, a subunit of the influenza A virus hemagglutinin glycoprotein HA, includes a hydrophobic peptide sequence which could facilitate lipid membrane destabilization^22^,^23^. These characteristics of AAV, TAT and HA2 spawned our idea that BDNF could be fused with the TAT and HA2 and then be packaged into AAV to generate the BDNF-HA2TAT/AAV which may be delivered intranasally as a novel anti-depressant agent.

The chronic mild stress (CMS) model was originally established by Katz and coworkers based on the etiology of depression^24^,^25^. This animal model consists of repeated exposures to a variety of mild stressors during a sustained time. This model has become a widely accepted rodent model of depression^26^. In rodents, the CMS program produced anhedonia expressed as reduction of the consumption of rewarding and palatable substances (mostly sucrose solution) which is also the core symptom of major depression^27^. Therefore, in our present study, the CMS model was employed to evaluate the intervening effects for depression-like behaviors of the designed BDNF-HA2TAT/AAV.

## Material and Methods

### Construction and Packaging of BDNF-HA2TAT/AAV

We design the BDNF-HA2TAT/AAV, and have it produced by Dr. Guang-xiao Yang as a previously reported with modification^28^. The AAV used in our present study are based on serotype 2. The AAV Helper-Free System (Stratagene) was used for viral vector preparation. Briefly, restriction enzyme sites (*Eco*RI and *Kpn*I, at upstream and downstream, respectively) were added in the BDNF cDNA by using PCR. The product was linked to Plasmid pGEM-T Easy which was then transferred into recipient bacterium (Top10 strain). The successfully transferred bacterial colony was selected by enzyme identification and was cultured to generate the BDNF cDNA with the added restriction enzyme sites. The DNA was extracted and linked to Plasmid PSSCMV-HA2TAT which was then transferred into Top10 strain. The correct BDNF-HA2TAT sequence was obtained and then transferred into Plasmid PUC19 for sequencing.

Three plasmids (pAAV/Ad, pAAV/Ad cofactor, pSSCMV-BDNF-HA2TAT) were transfected into HEK293 cells to generate virus AAV/BDNF-HA2TAT. The virus was quantified by Fluorescence quantitative PCR and the BDNF expression was confirmed by means of immunocytochemistry.

### Animal

Both male and female C57 BL/6J mice (aged: 7 ± 1 weeks; average body weight: 20 ± 2 g) were used for experiment. All the mice were purchased from Vital River Laboratories (Beijing, PR China). Mice, except the ones undergoing CMS procedure, were reared 4 per cage under a 12 h light/dark cycle (lights on from 7:00–19:00) and free access to food and water. All experimental procedures were approved by the Animal Care and Use Committee of Xi’an Jiaotong University. All animals were kept and the experiments were performed in accordance with the European Community guidelines for the use of experimental animals (86/609/EEC).

### Animal Experimental Scheme

The BDNF-HA2TAT/AAV was first applied to normal mice via different nasal dosage regimens to test its antidepressant efficacy. Based on the data, through the selected better regimen (intranasal administration for 10 days), the AAV was applied to mice subjected to CMS paradigm to verify its therapeutic effects. The behavioral tests were carried out 10 days after the last drug administration. Once the test finished, the CMS group mice were sacrificed, and the hippocampus brain region was separated on ice for BDNF western-blotting analysis.

**Figure 1:**
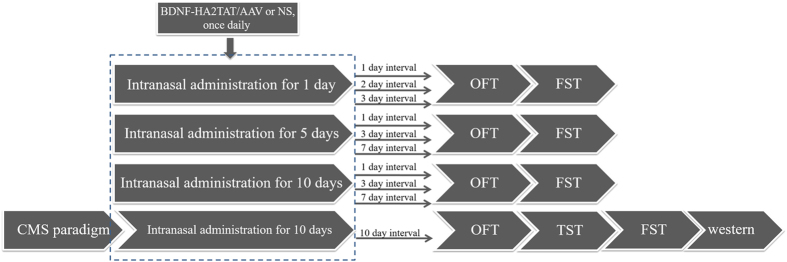
Flow diagram of the experiment arrangements. Mice (male/female) subjected to intranasal administration of BDNF-HA2TAT/AAV or normal saline (NS) for 1, 5, 10 days respectively. For each dosing regimen, behavioral tests (OFT and FST) were carried out certain days later as the figure showed. For the CMS group, male mice underwent intranasal delivery of BDNF-HA2TAT/AAV or NS for 10 days followed by behavioral tests (OFT, TST, and FST). Mice were sacrificed to obtain the hippocampus tissues, and Western-blotting analysis of hippocampal BDNF was performed afterwards. There was an interval of 1 day between the behavioral tests.

Male mice were divided into 20 groups, while female mice were divided into 18 groups (without CMS group). There are 8 mice in each group and the concrete arrangements are shown in the flow diagram (Fig. 1). The AAV/BDNF-HA2TAT or NS (normal saline) was delivered daily by nasal administration at the volume of 10 μl for each mouse (about 8.32 × 10^7^ genome copies of AAV vector) according to the titer test.

### Open Field Test (OFT)

Mice were placed individually into an open field chamber (45 × 45 × 45 cm) under a dim light (25 lx) for 1 hour and the tracks were recorded by a video tracking system (SMART, Panlab SL, Barcelona, Spain).

### Forced Swimming Test (FST)

Mice were placed into a Plexiglas barrel (15 cm diameter, 25 cm height) filled with water at room temperature (about 22 ± 1 °C) for 6 min. The mice were dried immediately and returned to the home cage. The immobility time was recorded by an observer without knowing the mice groups.

### Tail Suspension Test (TST)

The mice were suspended with a tape 0.75 cm away from their tails and elevated 70 cm above the floor. An observer who did not know the mice groups scored the immobility time for 6 minutes by visual observation.

### Sucrose Consumption Test (SCT)

Mice were deprived of water and food at the night before the test for 14 h. The sucrose was dissolved with distilled water at the concentration of 1% (mass/volume). The test was carried out in the next morning. The mice were provided with the sucrose solution during one hour period and mass difference value of the solution was measured.

Before the CMS started, 1% sucrose solution consumption baseline of the mice was measured for 3 times per week (Mondays, Wednesdays and Fridays) and lasted for 3 weeks when a stable baseline emerged (data not shown).

When the CMS procedure began, the SCT was performed in the morning every Wednesday.

### CMS Regimen

CMS group (n = 8) mice were housed individually in cages (26 cm × 18 cm × 13 cm) and received a battery of stress, including cage tilting, cage switching, wet cage, and inversion of light-dark cycle. The specific stress protocol is illustrated in Table 1.

To identify whether the CMS model was successfully established, a group of mice (n = 8) was employed as CMS control group. These mice were reared in the same condition but without any stress mentioned above. Only the SCT was applied to the mice in this group and the data obtained were used to compare with the CMS group.

### Western-blotting Analysis

Hippocampus tissues were obtained as described above. Western-blotting was performed according to a previous study^29^. The antibodies for BDNF (1:1000 dilution) and β-actin (1:5000 dilution) were purchased from Sigma -Aldrich, USA. The Quantity One software (Bio-Rad, Hercules, CA, USA) was used for quantification analysis.

### Statistical Analysis

All data were analyzed using SPSS16.0 software and expressed as mean ± standard error of mean (SEM). The data, except the SCT data which was analyzed by repeated measurement test, were analyzed with analysis of variance (ANOVA). P values that less than 0.05 were considered statistically significant.

## Results

### The Construction and Packaging of BDNF-HA2TAT/AAV

**Figure 2:**
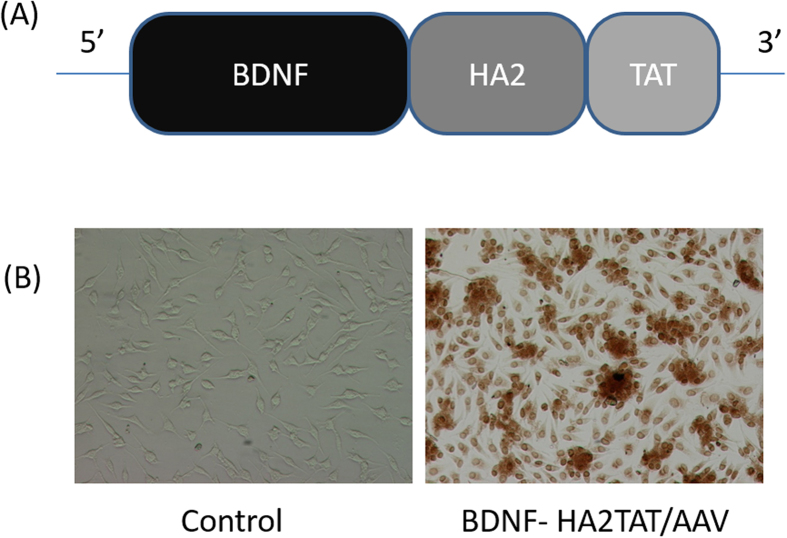
Construction and identification of BDNF-HA2TAT/AAV (**A**) The schematic figure which shows the construction of BDNF-HA2TAT/AAV. The HA2 (20aa) and TAT (11aa) are fused together and the sequence is ‘GLFEAIEGFIEGGWEGMIDGYGRKKRRORRR’. They are linked to the 3′ end of BDNF (murine, NCBI Reference: NP_001041604.1). (**B**) Immunocytochemistry result of Hela cells infected by the BDNF-HA2TAT/AAV. Compared with the control ones, the infected Hela cells showed high expression of BDNF.

The schematic structure of BDNF-HA2TAT is shown in Fig. 2A. Every step of BDNF-HA2TAT fusion gene construction was qualified by specific restriction enzyme reactions followed by agarose gel electrophoresis (AGE). By using the Sanger sequencing, the fusion BDNF-HA2TAT gene was identified. The sequence was identical as the designed one (data not shown).

The BDNF-HA2TAT/AAV virus was quantified by Fluorescence quantitative PCR and the titer was 8.32 × 10^9^ genomic copies/ml. Hela cells were infected by the AAV, and the BDNF expression was confirmed by means of immunocytochemistry (Fig. 2B).

### The Anti-depression Efficacy of BDNF-HA2TAT/AAV in Male and Female Mice

#### 1-day administration

**Figure 3:**
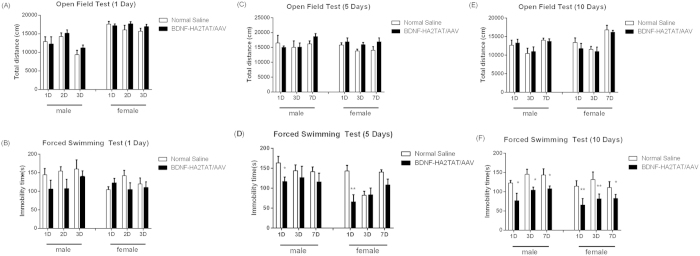
The Anti-depression Efficacy of BDNF-HA2TAT/AAV in Male and Female mice. (**A,B**) Behavioral tests after 1 day administration. The anti-depressant effects were measured after 1 day, 2 days or 3 days of the administration. (**A**) Open Field Test (OFT): Total distance moved in 1 hour. (**B**) Forced Swimming Test (FST): Immobility time. (**C,D**) Behavioral tests after 5 days administration. The anti-depressant effects were measured after 1 day, 3 days or 7 days of the last administration. (**C**) Open Field Test (OFT): Total distance moved in 1 hour. (**D**) Forced Swimming Test (FST): Immobility time. (**E,F**) Behavioral tests after 10 days administration. The anti-depressant effects were measured after 1 day, 3 days or 7 days of the last administration. (**E**) Open Field Test (OFT): Total distance moved in 1 hour. (**F**) Forced Swimming Test (FST): Immobility time. Data are shown as the means ± SEM. *p < 0.05, **p < 0.01.

Whenever the OFT and FST were performed (1, 3 or 7 days after administration), no statistical difference was found between the BDNF and NS groups of both genders (Fig. 3A,B, p > 0.05). However, it is mentionable that the FST immobility time of the BDNF groups of both genders generally showed a declining tendency.

#### 5-days administration

As shown in Fig. 3D, BDNF-HA2TAT/AAV treatment decreased the FST immobility time on the first day after the last nasal administration (p < 0.05). While this significant difference disappeared on the 3^rd^ or 7^th^ day after medication, the downtrend still remained. Meanwhile, the total distance of the OFT, indicating the horizontal locomotion, was not affected by BDNF-HA2TAT/AAV delivery (Fig. 3C, p > 0.05).

#### 10-days administration

When it comes to the situation that BDNF-HA2TAT/AAV was nasally administrated for sequential 10 days, compared to their control NS group, both the male and female BDNF group displayed significantly shorter FST immobility time (Fig. 3F, p < 0.05) without any differences in the total distance of OFT (Fig. 3E, p > 0.05).

### The Effects of BDNF-HA2TAT/AAV in CMS Mice

#### The sucrose consumption test

**Figure 4:**
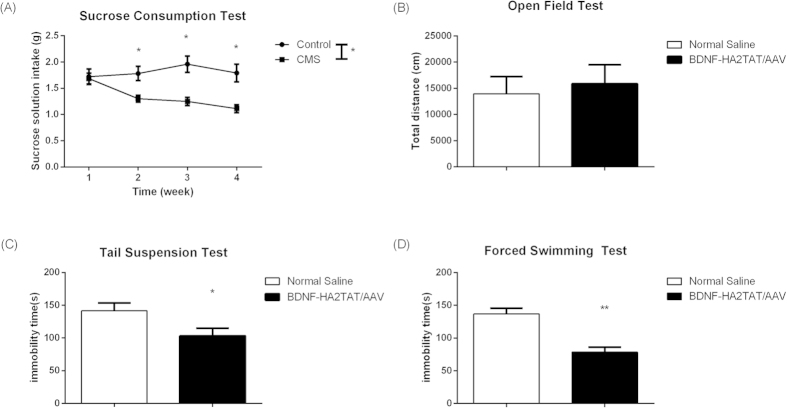
The Effects of BDNF-HA2TAT/AAV in CMS Mice. (**A**) The Sucrose Consumption Test (SCT): Compared to the control group, the sucrose solution intake gradually decreased due to the CMS onset. (**B–D**) Behavioral tests of the CMS mice after 10 days administration. The BDNF-HA2TAT/AAV was applied to mice subjected to CMS paradigm and the anti-depressant effects were measured after 10 days of last administration. (**B**) Open Field Test (OFT): Total distance moved in 1 hour. (**C**) Tail Suspension Test (TST): Immobility time. (**D**) Forced Swimming Test (FST): Immobility time. Data are shown as the means ± SEM. *p < 0.05, **p < 0.01.

As shown in Fig. 4A, compared to control group, the sucrose consumption of CMS mice decreased after the stress strategies began, indicating the core symptom of anhedonia of the CMS model was successfully derived.

#### The anti-depression effects after BDNF-HA2TAT/AAV administration

The BDNF-HA2TAT/AAV was applied to mice subjected to CMS paradigm and the anti-depressant effects were measured after 10 days of the last administration. While the total distance in OFT was not altered (Fig. 4B, p > 0.05), the AAV nasal treated mice revealed significantly decreased immobility time in both TST (p < 0.05) and FST (p < 0.01) compared to the NS control (Fig. 4C,D).

#### Western-blotting Analysis

**Figure 5:**
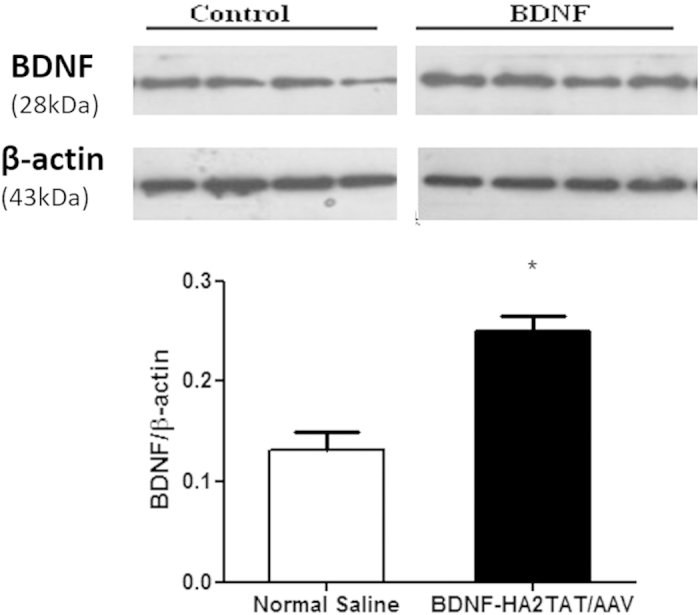
Western-blotting analysis. When the BDNF-HA2TAT/AAV was applied to the CMS mice, it obviously enhanced the BDNF level in hippocampus. The gels have been run under the same experimental conditions. The original blots are presented in the Supplementary Figure.

As shown in Fig. 5 (also Supplementary Figure), the BDNF expression in hippocampus was significantly enhanced in BDNF group CMS mice with comparison to that of NS ones after 10 days of treatment (p < 0.05).

## Discussion

The brain-derived neurotrophic factor (BDNF) has been investigated in several neuropsychiatric disorders including MDD, bipolar disorder, and addiction^30^. Especially with regard to depression, the decrement in neurotrophic factors (mainly BDNF) is a well-known pathogenesis hypothesis of depression^5^. Therefore, it is of great significance to explore the potential of BDNF protein as a novel anti-depressant agent.

However, delivering drugs into the CNS is always a challenge because of the impenetrable nature of BBB^31^. Several researchers attempted to apply BDNF for depression therapy by infusion^32^ or intracerebroventricular injection^33^ of the protein into rodents’ brain. Even though these methods break through the limitation of BBB and work well in the animal studies, the invasive nature of such administrations limits the application of these methods of BDNF delivery to clinical practice.

To find an approach to deliver BDNF effectively to the CNS for anti-depressant utility without tissue damage, we chose the nose-brain pathway as the administration route which provides a possibility to deliver drugs to the CNS bypassing the BBB^34^. The pathway of this delivery has not been clearly understood yet, with generally two hypotheses including the olfactory nerve pathway^35^ and the olfactory epithelium pathway^36^. Viruses and macromolecules such as protein possibly go through the first one which delivers the substance to olfactory bulb^34^. However, this approach has been demonstrated of slow rate of spreading with a time delay to reach the CNS^37^. This intriguingly explains the phenomenon in our present investigation that anti-depressive effects emerged only after relatively long duration of intranasal administration of BDNF-HA2TAT/AAV.

Besides, we embellished the BDNF with two cell-penetrating peptides (TAT and HA2) which could overcome a vital problem in utilizing proteins for therapy use that the protein alone is hard to pass through the cell membrane^38^. The main translocation mechanism of TAT is macropinocytosis^39^, while the HA2 could destabilize lipid membrane at low pH via an N-terminus insertion into membrane^22^. In addition, the fusion gene was packaged into AAV, a promising vector for gene transferring *in vivo*^40^,^41^. AAV-mediated intravitreal delivery of bFGF and BDNF can promote retinal cell survival following NMDA-induced excitotoxic insult^42^. In another study, it was found that BDNF, overexpressed via AAV delivery, could augment neurogenesis in both the normal and lesioned adult rat brain^43^. Intranasal routes for AAV for CNS delivery also have been attempted to deliver lysosomal enzyme to brain^44^. Besides, researchers have attempted to develop vaccination program of Alzheimer’s disease with the help of this mean of delivery^45^. Once administrated intranasally, the AAV intruded and colonized into the olfactory mucosa cells, consistently expressing the fusion gene and generating BDNF like a ‘small factory’. This advantage could probably benefit the patients with depression who have to take anti-depressants continually almost all-lifelong^46^. What’s more, this peculiarity could account for the prolonged anti-depressant effect observed in FST after the BDNF-HA2TAT/AAV delivery.

It is known that BDNF signals through its high-affinity tropomyosin-related kinase B (Trk B) receptor. Once activated, the BDNF-Trk B could regulate its downstream pathways including phospholipase C γ(PLCγ) pathway, the phosphatidylinositol 3-kinase (PI3K) pathway, and the mitogen-activated protein kinase (MAPK, or extracellular signal related kinase (ERK)) pathway^30^,^47^. It is also known that sustained levels of BDNF could down regulate the Trk B receptor. Previous study has demonstrated that continuous infusion of BDNF induced compensatory deactivation of Trk B, with total Trk B protein expression reduced by 70% after 6 days delivery^48^. In *a vitro* study, researchers also found that exposure of cultured hippocampal neurons to pharmacological doses of BDNF could lead to long-term down-regulation of trkB mRNA and TrkB receptor protein, resulting in dramatically decreased responsiveness to neurotrophin stimulation^49^. In our present study, the diminished immobility time of the FST existed only on the first day after 5-days administration. Besides, in the 10-days administration case, although the immobility time of all the BDNF-HA2TAT/AAV treatment groups were all shorter than that of their corresponding control groups, the significant difference downshifted as the interval after the last delivery increased. This phenomenon may not be due to the decline of the BDNF concentration, because the AAV could intrude and colonize into the olfactory mucosa cells, consistently expressing the fusion gene and generating BDNF as mentioned above. It is probably the compensatory deactivation of Trk B participated in. Besides, the dose of the vector delivered to the mice in current study is relatively lower which may be another potential reason.

After the BDNF-HA2TAT/AAV was successfully established, it was intranasally delivered to the mice via different dosing regimens as mentioned above. For all groups, there is no difference in the locomotor activity measured by OFT. This indicated that the potential anti-depressant, BDNF, has no mood-elevating effect. Besides, after receiving continuous administration of BDNF-HA2TAT/AAV via all regimens, the mice show no obvious health abnormality, indicating that the BDNF-HA2TAT/AAV is safe. For the part of its anti-depressive effect indicated by FST which was usually employed to investigate the efficacy of antidepressants^50^, no statistical difference was found in FST when the AAV was intranasally given to the mice only once. As the frequency of drug administration increased, the anti-depressive effect induced by BDNF-HA2TAT/AAV treatment became notable. What’s more, the female mice were more sensitive to the drug administration. As mentioned before, the prevalence of depression in females is much higher than that in males^4^. Therefore, the females may get more benefit throughout the treatment, which could explain the observed different intervening effects between male and female mice in our present study. In addition, the BDNF-HA2TAT/AAV was applied to the model mice of depression (CMS). The successful establishment was confirmed by SCT. Again, after BDNF-HA2TAT/AAV intranasally delivery, no mood-elevating effect was found in OFT, while the anti-depressive behavior was observed in both TST and FST. Further western-blotting analysis convinced that the hippocampal BDNF expression level was enhanced by BDNF-HA2TAT/AAV administration.

Indeed, there are some limitations in the present study which should be considered with caution. The hippocampal BDNF level indeed increased after drug treatment. However, whether the increased protein was delivered or endogenously produced is unclear. Considering the advantages of fluorescence labeling^51^, this technology may help to track the protein so as to identify its origin. Besides, our study was performed with mice. Extrapolation of the outcome to humans may be confounded by the species dissimilarities between the rodents and humans, such as nasal olfactory mucosa^52^ and physiology of the nose-brain pathway^53^. Therefore, further assessment should be performed before our finding could finally become an approach to depression therapy in human.

## Additional Information

**How to cite this article**: Ma, X.- *et al.* Intranasal Delivery of Recombinant AAV Containing BDNF Fused with HA2TAT: a Potential Promising Therapy Strategy for Major Depressive Disorder. *Sci. Rep.* **6**, 22404; doi: 10.1038/srep22404 (2016).

## Supplementary Material

Supplementary Information

## Figures and Tables

**Table 1:** The Chronic Mild Stress (CMS) Protocol.

	MON	TUE	WED	THU	FRI	SAT	SUN
Duration	9:00~19:00	7:00~19:00	9:00~10:00	9:00~19:00	7:00~19:00	9:00~11:00	7:00~19:00
Treatment	Cage tilting	Inversion of light-dark cycle	SCT	Cage switching	Empty cage	Group housing	Inversion of light-dark cycle
Duration	19:00 ~ next 7:00	19:00 ~ next 7:00	19:00 ~ next 7:00	19:00 ~ next 7:00	19:00 ~ next 7:00	19:00 ~ next 7:00	19:00 ~ next 7:00
Treatment	Inversion of light-dark cycle	Deprivation of water and food	Constant illumination	Cage tilting	Dirty cage	Inversion of light-dark cycle	Empty cage
