# Supplementary material for: Intranasal Delivery of Recombinant AAV Containing BDNF Fused with HA2TAT: a Potential Promising Therapy Strategy for Major Depressive Disorder
Source: Sci Rep. 2016 Mar 3;6:22404. doi: 10.1038/srep22404 (PMC4776097; doi:10.1038/srep22404)
Supplement: Supplementary Information [file srep22404-s1.pdf]

Intranasal Delivery of Recombinant AAV Containing BDNF Fused with HA2TAT: a Potential Promising Therapy Strategy for Major Depressive Disorder

Xian-cang Ma[1, a], Peng Liu[2, a], Xiao-ling Zhang[3, a], Wen-hui Jiang[1], Min Jia[1], Cai-xia Wang[4], Ying-ying Dong[1], Yong-hui Dang[2, 5, 6*], Cheng-ge Gao[1*]

[1]Department of Psychiatry, First Affiliated Hospital of Xi'an Jiaotong University Health Science Center, Xi'an, China

[2]College of Medicine & Forensics, Xi'an Jiaotong University Health Science Center, Xi'an, China

[3]Department of CT/MRI, Shaanxi Provincial People's Hospital, Xi'an, China

[4] Xi'an Ankang Hospital, Xi'an, China

[5]Key Laboratory of the Health Ministry for Forensic Medicine, Xi'an Jiaotong University Health Science Center, Xi'an, China

[6]Key Laboratory of Environment and Genes Related to Diseases of the Education Ministry, Xi'an Jiaotong University Health Science Center, Xi'an, China

[a] The first three authors contribute equally to this study

[*] **Corresponding author:**

Cheng-ge Gao, Department of Psychiatry, First Affiliated Hospital of Xi'an Jiaotong University Health Science Center; Xi'an 710061, China

E-mail: yaogaow@163.com

Yong-hui Dang, College of Medicine & Forensics, Xi'an Jiaotong University Health Science Center, Xi'an 710061, China

E-mail: psydyh@mail.xjtu.edu.cn

Supplementary Figure

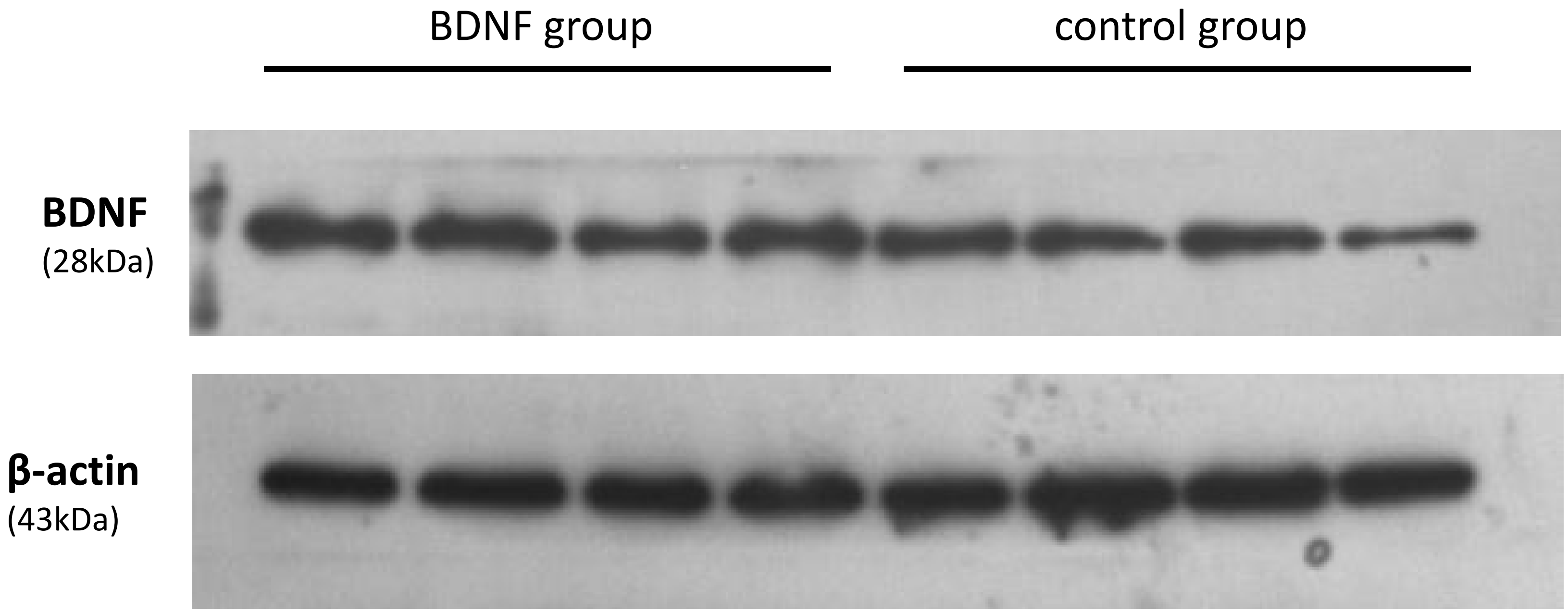

Supplementary Figure: Western blots showing the hippocampal BDNF expression level in the BDNF group CMS mice and the control mice.

Note: The gels have been run under the same experimental conditions. The membrane was cut according to the molecular weight range indicated by the marker. And then the membranes were incubated with the corresponding anti-bodies.
